# Plant-Derived Nano and Microvesicles for Human Health and Therapeutic Potential in Nanomedicine

**DOI:** 10.3390/pharmaceutics13040498

**Published:** 2021-04-06

**Authors:** Mariaevelina Alfieri, Antonietta Leone, Alfredo Ambrosone

**Affiliations:** Department of Pharmacy, University of Salerno, 84084 Fisciano, Italy; m.alfieri@santobonopausilipon.it (M.A.); aleone@unisa.it (A.L.)

**Keywords:** plant-derived nano and microvesicles, extracellular vesicles, EV biogenesis and uptake, nanomedicine, natural products

## Abstract

Plants produce different types of nano and micro-sized vesicles. Observed for the first time in the 60s, plant nano and microvesicles (PDVs) and their biological role have been inexplicably under investigated for a long time. Proteomic and metabolomic approaches revealed that PDVs carry numerous proteins with antifungal and antimicrobial activity, as well as bioactive metabolites with high pharmaceutical interest. PDVs have also been shown to be also involved in the intercellular transfer of small non-coding RNAs such as microRNAs, suggesting fascinating mechanisms of long-distance gene regulation and horizontal transfer of regulatory RNAs and inter-kingdom communications. High loading capacity, intrinsic biological activities, biocompatibility, and easy permeabilization in cell compartments make plant-derived vesicles excellent natural or bioengineered nanotools for biomedical applications. Growing evidence indicates that PDVs may exert anti-inflammatory, anti-oxidant, and anticancer activities in different in vitro and in vivo models. In addition, clinical trials are currently in progress to test the effectiveness of plant EVs in reducing insulin resistance and in preventing side effects of chemotherapy treatments. In this review, we concisely introduce PDVs, discuss shortly their most important biological and physiological roles in plants and provide clues on the use and the bioengineering of plant nano and microvesicles to develop innovative therapeutic tools in nanomedicine, able to encompass the current drawbacks in the delivery systems in nutraceutical and pharmaceutical technology. Finally, we predict that the advent of intense research efforts on PDVs may disclose new frontiers in plant biotechnology applied to nanomedicine.

## 1. Introduction

Eukaryotic cells secrete a large variety of vesicles under different physiological and pathological contexts. The biological role of extracellular vesicles (EVs) has been for a long time undervalued, assuming that the primary role of EVs is to serve as cell garbage bags collecting useless or harmful intracellular materials to be dismissed [1]. A large body of evidence is demonstrating, instead, that EVs do have an essential function as smart bioshuttles of lipids, proteins, nucleic acids, and other metabolites, and are naturally conceived to target cells by driving cell–cell communication, even between different organisms and species, thus regulating diverse biological and physiological processes [2,3,4].

Plants fabricate different types of nano and microvesicles (50–500 nm), localized in extracellular space or in the cytoplasm, whose function has been studied deeper only in the last decade.

In particular, proteomic approaches have shown that plant EVs carry numerous proteins with antifungal and antimicrobial activity, suggesting an important role in plant defense. Furthermore, it has been demonstrated that plant EVs are also involved in the intercellular transfer of small non-coding RNAs such as microRNAs, indicating the existence of mechanisms of gene regulation not yet explored and described in plants [5]. Plant EVs also participate in the cell wall remodeling by transporting important enzymes involved in the carbohydrate metabolism to the extracellular environment [6].

Several studies have been focused on the effects of nano (NV) and microvesicles (MV) derived from edible plants on human health and have been recently reviewed by several authors [7,8,9,10]. In particular, recent works have shown that plant-derived nano and microvesicles (PDV) extracted from citrus fruits and ginger roots have powerful anti-inflammatory and antineoplastic activities. Additionally, clinical trials are currently in progress to test the effectiveness of plant EVs in reducing insulin resistance, in controlling chronic inflammatory diseases and in preventing some side effects of chemotherapy treatments.

Additionally, the growing body of evidence on the main function of PDVs in the intercellular transport of biomolecules suggests their potential use as innovative carriers for the controlled release of drugs. Besides being highly biocompatible, it has been shown in different experimental models that they are excellent delivery tools of chemotherapeutics, overcoming some limitations of other delivery systems currently used in pharmaceutical technology.

In this review, we aimed not only at providing a general overview of the reported function of plant EVs on human health, but also to shed light on the research ongoing on plant-derived nano and microvesicles, underlining the knowledge gaps with respect to EV research in animal models.

## 2. Purification, Identification, and Classification of Plant-Derived Vesicles

The existence of nano-sized vesicles in plants has been reported since the second half of the last century, even before the mammalian exosomes discovered in 1983 [11,12]. 

In particular, ultrastructural analyses of cell clumps in suspension cultures of wild carrots demonstrated the presence of numerous multivesicular bodies able to fuse with the plasma membrane and to release their content into the wall space [13]. A few years later, membrane-bound vesicles were found in the microfibrillar material forming the cell wall apposition of tobacco leaves inoculated with non-pathogenic *Pseudomonas pisi* [14]. A similar observation were reported in barley leaves exposed to the biotrophic powdery mildew fungus *Blumeria graminis* f. sp. hordei [15].

However, PDVs have been poorly investigated and their biological role remains still not fully understood. This knowledge gap compared to what is currently known on EVs from mammalian cells, which are easily isolated from physiological fluids collected from the body (urine, blood, sweat, saliva, etc.) is probably due to the difficulty to purify plant EVs from extracellular fluids in most of the plant species, unless destructive procedures are employed.

**Figure 1:**
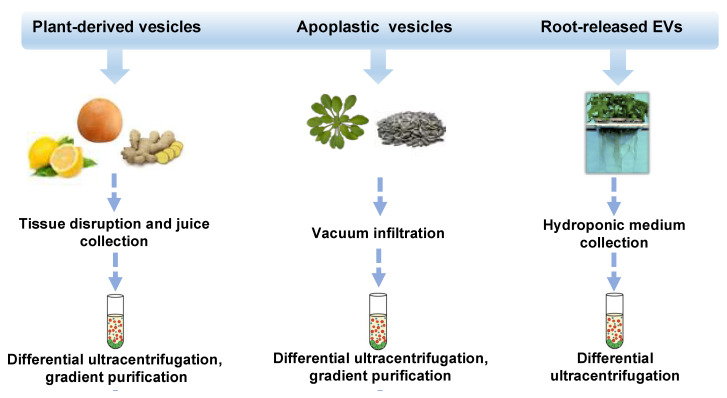
Isolation strategies and classification of plant-derived nano and microvesicles (PDVs). Overview of isolation methods and main types of plant vesicles (general plant-derived vesicles, apoplastic vesicles, and root-released vesicles) based on the plant source from which they derive (fruits or rhizomes, seedlings or seeds, and roots). Plant vesicles could be isolated trough differential ultracentrifugation steps and associated gradient purifications.

In the last decade, different approaches, mostly based on ultracentrifugation techniques, schematized in Figure 1, have been applied to isolate distinct classes of vesicles from tissues, organs, apoplastic fluid, and juices of several plant species such as ginger and carrot roots [2], grape [16] and citrus fruits [17,18,19,20], ginseng [21], apple [22], wheat [23] and many others. Very recently, round-shaped EVs were isolated by differential ultracentrifugation of a sampling solution containing root exudates of hydroponically grown tomato plants [24]. Nanovesicles have also been purified from the apoplast, which includes all the extracellular compartments, the cell wall, the xylem, and gas-and water-filled intercellular spaces. Thus far, apoplastic vesicles have been described in *Arabidopsis thaliana* [25] and *Helianthus annuus* [26].

The ultracentrifugation steps necessary for PDV purification are generally adapted from mammalian EV protocols, where biological fluids or cell culture media are mostly subjected to multiple steps of centrifugation. Basically, preliminary low-speed steps (300–16,000 *g*) are aimed at removing cells, debris, and large vesicles from the sample, while high-speed steps ranging from 100,000 to 200,000 *g* for 1–3 h are used to precipitate EVs. It is important to underline that these steps may vary according to the experimental conditions, biological samples, and laboratory expertise [27,28].

However, a unified protocol still not exist in plants as well; in fact some authors collect EVs by using lower speed centrifugation steps (ca 40,000 *g*), as described in *Arabidopsis* [25]. On the other side, many other protocols employ high-speed steps ranging from 100,000–150,000 *g*, for instance as reported in tomato, strawberry, garlic [24,29,30].

Alternative methods for EV isolation such as ultrafiltration, chromatography, polymer-based precipitation, and affinity capture on antibody-coupled magnetic beads are not common in plants [29].

It is noteworthy that the major limit of ultracentrifugation protocols relies on the copurification of contaminants including protein complexes and aggregates. To reduce the impact of undesired molecules on subsequent PDV use and to obtain more homogeneous vesicle populations, density-gradient purification can be applied downstream the ultracentrifugation steps [17,31,32]. Isolation strategies and subsequent physico-chemical characterization methods of plant extracellular vesicles are critically reviewed by Rutter and Innes [30].

The variety of vesicle populations isolated from plant tissues and organs together with the difficulty to distinguish them by using current isolation and characterization procedures has created some uncertainness regarding the nomenclature, reflecting the vexing problems of EV classification tackled for a long time by the International Society for Extracellular Vesicles [32,33].

Overall, it appears reasonable to adopt the terms “plant-derived vesicle” or “plant nanovesicles/microvesicles” when the extracellular origin of vesicles cannot be established, for instance in the case of vesicles obtained from juices or grinded plant tissues. In fact, in these cases, the preparations contain both intracellular and extracellular released vesicles. On the other side, terms like plant extracellular vesicles (EVs) can be given to all those vesicles extracted from extracellular compartments (e.g., apoplastic fluid, growth medium), as reported in *Arabidopsis*, sunflower, and tomato [24,25,26].

Finally, the term exosome, widely adopted in animal biology, is barely used in plants, primarily due to the absence or limited knowledge of specific protein markers of plant exosomes. Plant EVs carrying the tetraspanin 8 (TET8) and the mammalian homolog of exosome membrane protein CD63 have been described, suggesting their chemical and physical similarities with genuine exosomes [33]. In the absence of exosome markers, some authors used to name plant vesicles as exosome-like nanoparticles [34,35]. It is important to underline that the current knowledge is not yet sufficient to establish whether different kinds of PDVs may have different biological activity and efficacy when used for therapeutic purposes. Therefore, in the absence of clear information regarding their origin, in this review we use the general term PDVs to refer to plant nano and microvesicles, originated from either intra- or extracellular compartments.

### Biogenesis and Physico-Chemical Features of Plant-Derived Vesicles

**Figure 2:**
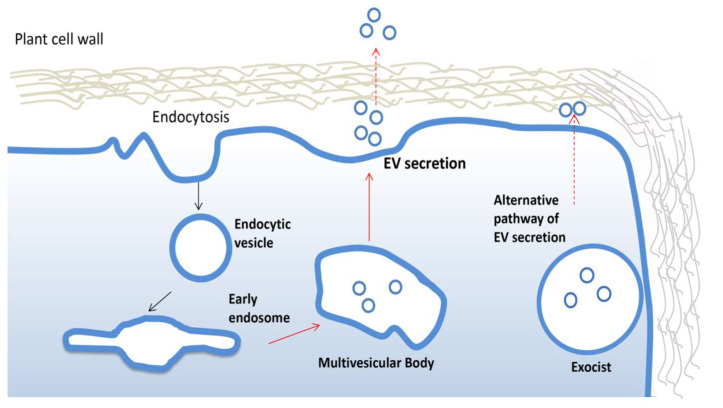
Biogenesis and secretion of plant extracellular vesicles. Plant extracellular vesicles (EVs) derive from early endosomes originated from plasma membrane by endocytosis. An early endosome becomes late endosomes and then forms multivesicular bodies which fuse with the membrane to release EVs. The alternative exocyst-mediated EVs secretion pathway described in plant is also shown.

PDV are often highly heterogeneous and contain fractions of intracellular vesicles and, therefore, it is not always possible to track the biogenesis. The formation and secretion of pure extracellular vesicles require multistep cellular processes well documented in animals [36]. Similar mechanisms have been described in plants (Figure 2). For instance, growing evidence suggests that vesicles destinated to extracellular space accumulate as intraluminal vesicles (ILVs) in plant multivesicular bodies (MVB) and are released by the fusion between MVBs and the plasma membrane. The MVB pathway is central for the turnover of transmembrane proteins and in eukaryotic cells [37]. The endosomal sorting complex required for transport (ESCRT) is the most important multi-protein complex responsible for the formation of MVBs and ILVs. It works as an ubiquitin-dependent mechanism, which binds and sequesters ubiquitinated proteins and sorts them into the ILVs of multi-vesicular bodies [38]. The ESCRT machinery is well conserved in plants, although ESCRT-0, involved in the initial targeting of the ubiquitinated cargo and the recruitment of the downstream ESCRT components, has not yet described in plants. Members of TOM1-like (TOL) protein family has been suggested as a plant-specific functional substitute for the ESCRT-0 in the initial targeting of ubiquitinated plasma membrane proteins destined for degradation [39]. However, more efforts are needed to uncover potential plant ESCRT genes and eventually establish the contribution of each component of this pathway in plant EV biogenesis, as already described for mammalian EVs.

Interestingly, an alternative mechanism to ESCRT has been described for the biogenesis and release of EVs in plant. In particular, in different plant organs, the presence of a spherical cell compartment enveloped by a double membrane, denominated EXPOs (exocyst positive organelles), has been reported. Colocalization analyses with endocytic and MVB markers proved that EXPOs are distinct entities from endocytic vesicles. Moreover, EXPOs are able to fuse with the plasma membrane and release a single membrane vesicle into the cell wall [40].

It is also worth mentioning that PDVs may reside in the paramural space or be secreted outside the cell wall (EVs). How plant EVs cross the cell wall and reach the extracellular space is another intriguing aspect not fully understood so far. Indeed, the pore size of cell walls are smaller than the EV mean diameter, building a natural barrier not easily crossed by EVs. Although not yet proven, enzymes carried by EVs, already known for their involvement in the remodeling of the cell wall, could determine a transient destabilization of this external structure, facilitating their passage through the cell wall and successful releasing of the EVs in the extramural space. Alternatively, it has been suggested that reversible-stretching of plant cells wall determined by its natural plasticity or local breaks may help EVs to pass through it.

**Figure 3:**
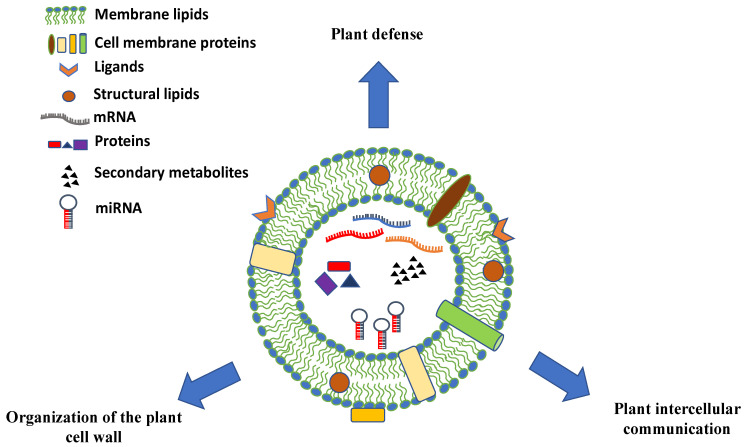
Schematic representation, composition, and biological roles of plant-derived vesicles. Plant vesicles are round-shaped nano and microstructures containing a vast array of proteins, nucleic acids (mRNAs, miRNAs, and other types of short RNAs), and secondary metabolites surrounded by a lipid bilayer with membrane proteins, channels, ligands, and receptors.

The chemical composition of PDVs can vary greatly and give hints on the biological role of these vesicles in plants. PDVs have a high percentage of lipids whose composition and assembly seem to be very important for understanding the uptake mechanisms of the vesicles themselves. In particular, in many vesicles there is the presence of phosphatidic acid (PA), which appears to be involved in the membrane fusion mechanisms [41]. In addition to lipids, plant PDV carry distinct classes of biomolecules. They are rich in proteins that may play specific functions, such as proteins involved in the defense against pathogens or in response to biotic and abiotic stress and enzymes involved in the synthesis of the cell wall (Figure 3). Moreover, many EV surface proteins are transmembrane proteins, often derived from MVB or from the trans-Golgi network (TGN), which are used as molecular markers for their identification [42]. PDVs also contain small RNAs and miRNAs molecules that appear to participate in the intercellular communication between cells of organisms belonging to different species or to different kingdoms [43].

Before reviewing in detail the biological properties and the pharmacological potential of PDV components, in the next paragraphs the native role of PDVs and their implications in plant biological processes will be briefly summarized and discussed.

## 3. Biological Functions of Nano and Microvesicles in Plants

Although knowledge is still limited, recent studies have shown that the plant EVs may play multiple roles in plants. The involvement of EVs in plant immune responses and in response to biotic and abiotic stress has been clearly demonstrated over the last decade. Furthermore, they may also be involved in the reorganization of the cell wall and in intercellular communication, also among species belonging to different kingdoms.

### 3.1. Role of EVs in Plant Defense

It is well documented that plant nano and microvesicles have an active role against pathogens. Since the first observations, MVBs were proved to proliferate in plant cells during the pathogen attack and to fuse with the plasma membrane close to papillae, extracellular defense structures that reinforce the cell wall, preventing pathogen cell invasion [15,44,45]. The proteome of leaf apoplastic vesicles purified from *A. thaliana* plants is enriched in proteins involved in signal transmission in response to biotic and abiotic stress and proteins contributing to acquire immunity. Moreover, *A. thaliana* EV secretion is significantly enhanced during *Pseudomonas syringae* infection or upon elicitation with salicylic acid [25]. EV isolated from extracellular fluids of sunflower seedlings contain high levels of cell wall remodeling enzymes and defense proteins and are able to enter the phytopathogenic fungus *Sclerotinia sclerotiorum,* thus impairing fungal spore growth and development [46].

Recently, shot-gun proteomic analysis of plant EVs purified from root exudates of tomato plants have revealed the presence of numerous proteins known to be involved in plant-microbe interactions such as endochitinases and glucan-endo-1,3 beta glucosidase B precursors and putative late blight resistance protein homologs. Furthermore, root-released EVs were shown to inhibit in vitro spore germination of the plant pathogens *Fusarium oxysporum*, *Botrytis cinerea*, and *Alternaria alternata* [24].

Further studies have shown that EVs can be tools for delivering genetic information and can mediate a new type of communication between organisms belonging to different kingdoms. A recent fascinating work in *Arabidopsis* has clearly demonstrated that EVs can modulate gene expression of the infecting *Botrytis cinerea* through the horizontal transfer of small RNAs (sRNAs) and miRNAs to the pathogen, thus inhibiting the mRNA translation, and in this way limiting its pathogenicity. In particular, the authors showed that these vesicles contain sRNAs such as TAS1c-siR483 and TAS2-siR453 capable of repressing the expression of the fungal genes C1G10728, BC1G_10508, and BC1G_08464, all involved in vesicular trafficking pathways and used by *B. cinerea* as virulence factors [33]. Altogether, these data provide a strong support to the hypothesis that plant EVs could be new components of the plant innate immune system.

### 3.2. Role of Plant EVs in Intercellular Communication

While the role of EVs in intercellular communication is well consolidated in the animal kingdom, advances in plant EVs are still behind. The finding of plant apoplastic proteins without canonical signal peptides in their coding sequences suggests the existence of unconventional protein secretion mechanisms (UPS), independent from ER/Golgi trafficking, as already widely reported in other eukaryotes [47]. Proteomic studies conducted on EVs released from sunflower seeds (*Helianthus annuus*) have shown the presence of high concentrations of Helja, a lectin devoid of the signal peptide, confirming the existence of UPS mechanisms mediated by vesicles [26]. These mechanisms may permit the secretion of many proteins, bypassing the trafficking networks of ER/Golgi [48]. Intriguingly, it has been reported that many proteins devoid of signal peptides are involved in the response to pathogens and environmental stress [49], supporting the hypothesis that plant EVs represent important mediators of protein secretion, especially in conditions of adverse growth or in the presence of pathogen attacks.

Finally, a recent study has shown that *Arabidopsis* EVs are able to carry a new class of sRNA, namely *tiny RNAs*, owing to their small size (10–17 nucleotides). At the present, it is not yet clear whether these RNAs have a specific cellular function or whether they are a product of cellular metabolism. They could be involved in the regulation of gene transcription, probably activating the expression of specific genes with a mechanism similar to that described for small activating RNAs (saRNAs) in mammals. EVs, carrying tiny RNAs, might play an important role in the transfer of genetic information in the plant [5]. EVs have also been identified in the phloem of plants, a tissue that transports sugars to metabolically active or accumulation (sink) tissues. The localization and diffusion of EVs in a strategic tissue such as the phloem provides further indications to support their important role in intercellular communication in plants.

### 3.3. Role of EVs in the Organization of the Plant Cell Wall

Plant EVs may also accumulate in the extracellular space delimited by the membrane and the cell wall (intramural vesicles), where they may have a potential involvement in wall remodeling. In particular, a proteomic study highlighted that about 47% of the proteins identified in the EVs-enriched fraction from sunflower (*Helianthus annuus*) apoplastic fluids are proteins related to the cell wall [6]. Sunflower EVs show an enrichment of proteins associated with carbohydrate metabolism. Many of these proteins such as glycosyl hydrolases (GH), expansins, and arabinogalactan proteins are already known to be involved in the degradation and reorganization of the cell wall. Other important proteins such as proteases, Kunitz-inhibitors, peroxidases, GDSL lipases, germin-like proteins, lipid-transfer proteins, and multicopper oxidases normally associated with cell wall activities have been identified in sunflower EVs. Cell wall-related proteins were also identified in Arabidopsis apoplastic vesicles [25].

Although the current knowledge of plant extracellular vesicles is still in a preliminary stage, these results strongly support the notion that EVs actively take part in the remodeling of the plant cell wall. In addition, plant EVs could act in important biological processes which require the contribution of the outer wall, such as those related to development/growth of the plants.

## 4. PDVs Carry Diverse Bioactive Molecules with High Pharmaceutical and Nutraceutical Interests

The composition of plant PDVs varies according to their biological source. Hence, the variegate cargo composition of these vesicles allows us to explain, at least in part, the mechanisms of action, at least in part, behind the multifaceted therapeutic effects described so far. Lipids, proteins, nucleic acids, and metabolites are major components of PDVs (Figure 3). However, wide-spectrum analyses of PDV biomolecules have been reported only for a few plant species. Therefore, further omics efforts are strongly necessary in order to unveil the biologically active natural compounds delivered by PDVs.

### 4.1. Lipids

Lipids are essential constituents of EVs. Despite their important role in EV formation and bioactivity, knowledge about the lipid composition and function of plant vesicles is still limited [50]. Basically, lipidomic analyses of EVs conducted in animal cell lines reported that the lipid patterns of EVs are different from that of their cells of origin, suggesting a selective lipid loading mechanism for EVs [51]. Overall, EVs are rich in ceramide and phosphatidic acids, which are both lipids critical for exosome formation [52,53].

Lipidomic analysis of *Arabidopsis* rosette leaf EVs revealed a great percentage of sphingolipids (~46%), much higher than that found in whole leaf tissues. Glycosylinositolphosphoceramides (GIPCs), a particular class of sphingolipids, accounted for up to 99% of the total sphingolipids found in EVs. The high enrichment of GIPCs in plant EVs is suggestive of a signaling function of the EV membrane, especially in the extracellular ROS burst, as proven in *Arabidopsis* plants [54]. However, possible biological implications of plant EV sphingolipids have not yet been investigated in mammalian cells.

Lipidomic profiles of orange NVs (ONVs) showed that these vesicles contained phosphatidylethanolamine (PE) (~40%), phosphatidylcholine (PC) (~25%), phosphatidylinositol (PI), (~12%) and phosphatidic acid (PA) (~5%) [55]. Similarly, grapefruit-derived vesicles revealed an enrichment in phosphatidylethanolamine (45.52%) and phosphatidylcholine (28.53%). PDVs of grapes, sunflowers, and ginger contain phosphatidic acid (PA) as well [10]. Besides playing an important role in the mechanism of fusion of the membranes and in the release of these vesicles, PA is as an important class of lipid messengers involved in many cellular processes such as cytoskeletal organization, cell proliferation, and survival [56]. Other studies have shown that phosphatidylcholine of ginger EVs plays an important role in the migration from the intestine, where they accumulate following intake into the liver [57]. Not least, therapeutic addition of PC to the colonic mucus may alleviate the inflammatory activity, indicating that part of the beneficial effects of PDV in the gastrointestinal tract may be attributed to the significant presence of PC [58]. It has also been shown that plant Evs are able to modify the composition and localization of intestinal microbiota bacteria and that lipids determine selective PDV entry in bacteria species. For example, the presence of phosphatidic acid is fundamental to drive the entry of ginger-derived vesicles in *Lactobacillus rhamnosu*, and phosphatidylcholine is essential for the uptake of grapefruit-derived vesicles by the bacterial family of *Ruminococcaceae* [57]. *A* further functional evidences of bioactive lipids came from ginger exosome-like nanoparticles (G-ELNs). In particular, total lipids extracted from G-ELNs were dried, reassembled into liposomes, and then proven to suppress the NLRP3 inflammasome activity in bone marrow-derived macrophages [59].

### 4.2. Proteins

Exosomal proteins are extensively studied, as they serve as diagnostic biomarkers, and also for the functional role they may play in the intercellular communication [60]. Plant EV proteins are mainly represented by transmembrane and plasmalemma-associated proteins, although specific markers have not been clearly identified so far. Ginger-derived EVs contain a low protein content, predominantly composed by cytosolic proteins (e.g., actin and proteolysis enzymes) and a few membrane channel/transporters (e.g., aquaporin and chloride channels) [61]. It was reported that protein composition of grape-derived exosomes is mainly characterized by metabolism enzymes, aquaporin proteins, but also chaperones such as HSP70 [16]. The EVs proteome of four different *Citrus* species showed the presence of highly expressed HSP70, HSP80, 14-3-3, G3PD and FBA6, PTL3, and clathrin proteins. Aquaporin, different hydrolases (ATPases, pectinesterase, phospholipases, amylases, β galatosidases, and adenosylhomocystein hydrolyse), and enzymes (SODs, CATs, PODs, and GPXs) were identified [17]. Aquaporins have been reported to play an important role in vesicle stability in the case of plasma membrane vesicles purified from broccoli plants [62].

In Arabidopsis, EV proteome was highly enriched in proteins involved in biotic and abiotic stress responses. Several proteins involved in signal transmission were identified, many of which highly induced in response to stress and/or contributing to immunity, such as RPM1-INTERACTING PROTEIN4 (RIN4) and its interactors including ATPASE2 (AT4G30190), EARLY-RESPONSIVE TO DEHYDRATION4 (AT1G30360), REMORIN (AT3G61260), and DELTA (24)-STEROL REDUCTASE. These findings suggest that plant EVs may play a role in microbe-associated molecular pattern or effector-triggered immunity [25]. In Arabidopsis EVs were also found proteins involved in the myrosinase-glucosinolate system, in reactive oxygen species (ROS) signaling and oxidative stress responses, various membrane-trafficking proteins, and numerous proteins for the transport of ions, water, sugar, and other substrates. The presence of proton pumps suggests that EVs actively transport ions, regulating their own membrane potentials [63]. Although different studies reported information about the protein composition of plant EVs (metabolic enzymes, signal transduction factors, adhesion factors, cytoskeleton proteins, and ubiquitin), further investigations are needed to shed light on their potential use in pharmacological applications.

### 4.3. Nucleic Acids

EVs have also been shown to contain a variegated plethora of different types of nucleic acids (mRNA, miRNA, but also DNA). Among them, an increasing body of evidence is uncovering that miRNAs of edible plants could target genes in the mammalian genome and thus play a role in inter-kingdom communication [64]. Plant miRNAs are naturally modified at their 3′-ends with a 2′-*O*-methylation [65]. This chemical modification seems to protect plant miRNAs from degradation and uridylation, making them more stable than animal miRNAs in different environments [66]. Although experimental evidences are still controversial, plant miRNA have also been proven to play biological roles in target cells [67]. For instance, MIR168a from rice, commonly detected in serum of Chinese subjects, reduce the expression of the low-density lipoprotein receptor adapter protein 1 in mouse liver, and consequently decrease LDL removal from mouse plasma [68]. More recently, oral administration of plant-derived miR2911 has been shown to retard liver fibrosis in mice [69].

The mechanisms of plant miRNA uptake in animal cells are not yet fully understood, even though recent findings support that PDVs may deliver intact miRNAs to recipient cells. Teng and colleagues showed that ginger-derived exosome-like nanoparticles (GELNs) can favorably modulate the composition of gut microbiota, determining an increase in *Lactobacillaceae* and *Bacteroidales S24-7*, and a decrease in *Clostridiaceae* in GELN-treated mice in comparison with mice treated with PBS. Next-generation sequencing analysis of GELN RNA revealed that GELNs contained at least 109 mature miRNAs that could target numerous bacterial mRNA. In particular, in a mouse model of inflammatory bowel disease with DSS-induced colitis, ath-miR167a could bind the pilus protein SpaC mRNA and induce its downregulation in *Lactobacillus rhamnosus*, reducing its translocation into the peripheral blood and concomitantly increasing the permanence on mucosal surfaces. In addition, the gma-miR396e of GELNs promotes *L. rhamnosus* growth at least in part through the inhibition of LexA expression. Altogether, these data show how diet ELN miRNAs can cross-talk with gut microbiota to maintain gut health. Moreover, ginger RNA may modulate the production of the biologically active metabolite indole 3-acetaldehyde, synthesized by some species of the genus *Lactobacillus*, which stimulates the production of interleukin-22, a cytokine which improves the stabilization of the mucosa in cases of colitis [57].

Another study has shown that highly expressed miRNAs in PDVs, obtained from 11 edible plants, can potentially regulate the expression of inflammatory cytokines and cancer-related genes in vitro, potentially mediating interspecies intercellular communication [35]. In this study, miR-168c is likely involved in regulating the expression of the *TSC22D3* gene encoding a protein whose expression is stimulated by glucocorticoids. This protein plays a key role in the onset of the anti-inflammatory and immunosuppressive effects of these drugs; EVs could therefore influence the effects of glucocorticoids by regulating the expression of this gene and could have immunosuppressive properties.

Moreover, miR-156c and miR-159a, able to regulate mammalian TNF-α signaling pathway in adipocytes and regulate inflammation, were identified in the exosome-like nanoparticles derived from walnut plants [70]. A recent study demonstrated that EVs from different edible plants (soybean, ginger, hamimelon, grapefruit, tomato, and pear) contain miRNAs specifically targeting regions of SARS-CoV-2, but not SARS-CoV. In particular, osa/cme miR-530b-5p targeted the ribosomal slippage site between ORF1a and ORF1b [71]. However, further investigations are required in order to shed light on the intracellular stability of EV miRNAs and their proposed anti-viral activity under in vitro and in vivo conditions.

### 4.4. Plant Metabolites

Plants produce a wide range of bioactive molecules involved in primary and secondary metabolism [72,73]. As plant EVs have been shown to play a major role in plant protection, PDVs may represent a valuable source of bioactive compounds, whose functions need to be accurately evaluated in order to understand their biological effects on human health. Despite this consideration, at the present, metabolomic data of PDVs are still scarce. A recent metabolomics study revealed distinct metabolome profiles of the juice and vesicle fractions of grapefruit [74]. In particular, grapefruit NVs exhibited a high relative amount of amino acids (leucine/isoleucine) and organic acids (mainly glycolic and citric acids), whilst MVs and fruit juice were characterized by a high percentage of sugars and sugar derivatives. Other important bioactive compounds, such as quinic acid, myo-inositol, and aucubin were also found in grapefruit-derived vesicles. These molecules may provide multiple beneficial effects of grapefruit-derived nano and microvesicles described so far.

Differential metabolite profiles were also observed in orange juices and orange-derived nanovesicles (ONVs). In particular, ONVs were shown to contain carbohydrates (glucose, fructose, sucrose) and amino acids (alanine, asparagine isoleucine, threonine, leucine). ONVs were also characterized by significantly higher levels of leucine, threonine, formate, methanol, ethanol, and *sn*-glycero-3-phosphocholine with respect to orange juice. On the other side, ONVs did not contain vitamin C and naringenin, which are major active compounds of the orange juice. Despite these pioneering works, the knowledge of secondary metabolites contained in PDVs remain largely unknown, thus suggesting that this field of investigation needs to be strongly supported and implemented in the near future in order to disclose the diversity of PDV secondary metabolites and shed light on their contribution in PDV bioactivity [55].

## 5. Plant-Derived Vesicles for Human Health: From Cell Uptake to Therapeutic Potential

In the last few years, several lines of research have been focused on the potential benefits that EVs may have on human health and their possible use as natural or bioengineered carriers for the transport and release of pharmaceutical and/or nutraceutical compounds. Somehow, nanotechnological tools first evolved in cells. In fact, billion years of evolution made cell-derived vesicles outstanding nanotools for intercellular transport. Such nano-sized plant vesicles may be fully considered as new weapons for drug cellular targeting in nanomedicine. Before describing potential applications in this field, it is quite critical, however, to summarize what is currently known on the uptake mechanisms of PDVs in the target cells.

### 5.1. Uptake Mechanisms of Plant-Derived Vesicles in Mammalian Cells

EVs are structures naturally designed to transport molecules from one cell to another and to protect their cargo from enzymatic degradation occurring in the extracellular environment. The presence of EVs in bacteria, fungi, plants, and animals suggests that these functions are strongly conserved during the evolution. As mentioned above, numerous cases of EV exchange between cells of organisms belonging to different species have been documented, suggesting that these structures are able to penetrate into many types of cells and release their own content within them.

Overall, multiple direct visualizations of EV uptake have been reported by using EV labelled with fluorescent lipid membrane dyes and subsequent observations of EV-treated cells or organisms by fluorescence and confocal microscopy [75,76,77]. Routes and mechanism of EVs uptake in mammalian cells remain intensively debated in the literature, however, clathrin-mediated endocytosis, phagocytosis, macropinocytosis, and plasma or endosomal membrane fusion of EVs have been largely described, as exhaustively reviewed by Mulvahy and colleagues [78].

**Figure 4:**
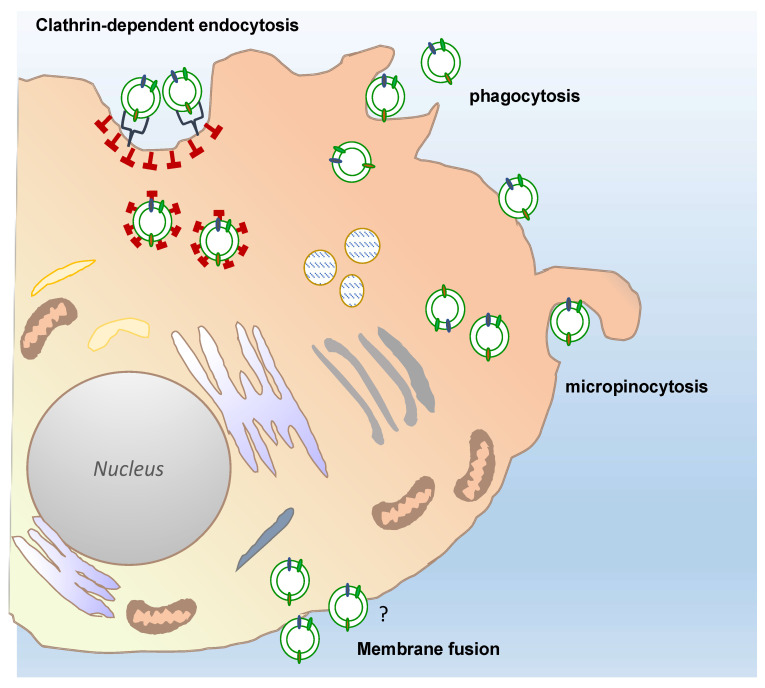
Possible different routes of plant-derived vesicles uptake into a target cell. Recipient cells can receive plant-derived vesicles through different, well described uptake mechanisms: Phagocytosis, macropinocytosis, and clathrin-dependent endocytosis. The passive membrane fusion is also shown in the figure. The question mark indicates that this mechanisms remains still to be elucidated for PDV uptake, although described for many types of EVs.

Only a few reports have so far described the uptake mechanisms of plant-derived vesicles in mammalian cells (Figure 4). The results of such studies have only partially shed light on the routes by which PDVs enter mammalian cells. For instance, confocal analyses of macrophages exposed to fluorescently labelled grapefruit EVs have shown that the EVs accumulated in the perinuclear region of the mammalian cells. The use of specific inhibitors targeting cell entry pathways also proved that grapefruit EVs were taken up through macropinocytosis and by clathrin-dependent endocytosis. Similarly, ginger EVs are internalized by macropinocytosis in hepatocytes, whereas cell uptake occurs prevalently through phagocytosis in colon cancer cells [79,80].

Recently, it has been elegantly demonstrated that PDVs may enter mammalian cells by using specific cell surface interactors. More in detail, the CD98 glycoprotein present on the HepG2 hepatocellular carcinoma cells drives the internalization of garlic-derived vesicles through the specific binding with lectin-type proteins located on the vesicle surface [81]. Taking into account that CD98 is highly expressed in many types of cancers as well as during gut inflammation and liver diseases, these finding disclose new molecular mechanisms of cell-PDV interactions that contribute to better understand the therapeutic potential of PDVs.

### 5.2. Application of Plant EVs for the Treatment and Prevention of Human Diseases

As an integral part of our diet, vesicles derived from fruits and vegetables need to be thoroughly investigated in order to understand potentially beneficial and eventually detrimental effects on human beings. Noteworthy, PDVs may be shuttled and reach the gastrointestinal tract as intact structures, participate in the renewal of intestinal tissue, and modulate the intestinal microbiota. In addition, PDVs have been proven to have beneficial functions against inflammatory diseases such as colitis and steatosis. Recent studies have also shown that these vesicles may also inhibit the proliferation of tumor cells of certain cellular lines, thus representing a new potential approach in cancer treatment. The most important findings regarding the use of PDVs to treat and/or prevent human diseases are summarized in Table 1 and briefly presented and discussed in the following paragraphs.

### 5.3. Role of PDVs in Bowel Diseases and Diet-Induced Dysfunctions

Several in vivo and in vitro studies have been conducted to understand the effects of nano and microvesicles from edible plants on the gut intestinal tract (GIT). EVs derived from grapes, grapefruit, and ginger have been demonstrated to contribute to the maintaining of the correct functioning of the intestine, favoring the process of renewal of enterocytes. In particular, grape exosome-like nanoparticles (GELNs) were gavage-administered to mice and detected intact in intestinal stem cells, demonstrating that PDVs might penetrate the intestinal mucus barrier. Moreover, application of GELNs increased the production of stem cells in intestinal epithelium in vivo as well as in three-dimensional intestinal culture systems. Finally, GELNs prevented dextran sulphate sodium (DSS)-induced colitis in mice, supporting their important biological implications in the intestinal tissue homeostasis and repair mechanisms [16].

Additionally, PDVs extracted from grapefruit (*Citrus paradisi*) are resistant to digestive enzymes such as pepsin and intestinal pancreatin and reach intact in the intestine where they are selectively absorbed by intestinal macrophages [82]. In particular, it was found that mice with DSS-induced colitis treated with grapefruit EVs had lower colonic shortening, reduced weight loss and a decrease in the severity of the pathology and local lymphocyte infiltration. Grapefruit EVs induced the expression of heme-oxygenase 1, an enzyme with important antioxidant properties, and of IL-10, an interleukin with anti-inflammatory and immunosuppressive properties. In addition, grapefruit EVs inhibited the production of pro-inflammatory cytokines by intestinal macrophages such as IL-1 β, TNF-α, and IL-6, and decreased the levels of chemokines which play an important role in the recruitment of monocytes, lymphocytes T, and inflammatory cells such as MCP-1, CXCL9, and CXCL10. In addition, they present high concentrations of phosphatidylcholine and phosphatidylethanolamine, lipids with known antioxidant and anti-inflammatory properties. Altogether, these data proved that grapefruit EVs play a key role in modulating the immune response of the intestine and reduce the inflammatory process in case of pathology [82].

PDVs derived from broccoli could be useful in the prevention of colitis as well. Broccoli-derived nanoparticles were shown to activate adenosine monophosphate-activated protein kinase (AMPK) in dendritic cells, thus regulating the homeostasis of the intestinal immune system [45]. PDVs extracted from the roots of the ginger (*Zingiber officinalis*) have been demonstrated to reduce the inflammatory process and the local lymphocyte infiltration in mice treated with DSS, to favor the repair of the intestinal epithelium, and also to prevent colitis-associated cancer [61].

Based on these results, PDVs could be considered in the treatment of chronic inflammatory diseases of the gastrointestinal tract as they promote the repair of the intestinal epithelium and the resolution of the inflammatory process.

Although PDVs can be considered active constituents of our diet, limited knowledge is available about their role as nutraceuticals. Recently, nutritional and metabolic aspects of orange derived nanovesicles (ONVs) were examined by using a high-fat, high-sucrose diet model (HFHSD mice) [55]. Firstly, the authors demonstrated that ONVs concentrate and deliver plant species-specific lipids and metabolites to artificial intestinal barriers and in the jejunum of HFHSD mice, namely in the intestinal region involved in dietary lipid absorption. Further, they investigated functional properties on the intestine in vivo demonstrating that ONVs induced an increase in villi size in HFHSD jejunum, modulated mRNA levels of genes involved in immune response and decreased jejunum triglyceride content during the fasting periods. High levels of phosphatidic and leucine in ONVs may account for their anti-inflammatory properties and improved glucose metabolism in obese mice.

### 5.4. Role of Plant EVs in Liver Disease

Fluorescently labelled-ginger-derived nanoparticles orally administered to mice were found to accumulate in liver with a peak intensity at 12 h and in mesenteric lymph nodes. Interestingly, the authors noticed that ginger EVs penetrate faster and into larger amounts in the liver of mice fed an ethanol-based diet that induces steatosis and fat accumulation in the liver, with respect to mice fed a regular diet and no liver damage. These results led to the conclusion that the extent of PDV absorption, besides the cell type, also depends on physio-pathological conditions [79].

In addition, mice treated for 7 days with 50 mg of ginger-derived nanoparticles showed a lower concentration of liver triglycerides, reduced size of the liver, and reduced infiltration of mononuclear cells, thus suggesting that these vesicles play a preventive role in hepatic steatosis. It was also shown that the ginger EVs are involved in the activation of nuclear factor erythroid 2–related factor 2 (*Nrf2*) that is able to activate the expression of a group of antioxidant and detoxifying liver genes and to inhibit the production of reactive oxygen species. This may be in part the protective action of ginger-derived vesicles toward alcohol-induced liver injury [79].

### 5.5. Antitumor Activity of Plant EVs

Recent studies have shown that the EVs derived from citrus fruits may inhibit the proliferation of cancer cells. For instance, vesicles extracted from lemon juice (*Citrus limon)* were tested on different tumor cell lines showing reduction of 50% of the growth and tumor progression 48 h post-treatment [83]. Interestingly, lemon-derived vesicles did not impair the growth of non-cancerous cell lines, demonstrating selective bioactivity in tumor cells. Lemon-derived vesicles increases the levels of mRNAs coding for pro-apoptotic proteins such as Bad and Bax and lower expression of anti-apoptotic genes such as survivin and Bcl-xl. Tumor necrosis factor-related apoptosis-inducing ligand (TRAIL) and its receptor DR5 were found to be up-regulated in EVs-treated tumor cells. Antineoplastic effects of EVs derived from lemon were also tested in vivo using a xenograft model of chronic myeloid leukaemia. Interestingly, EVs injected into peritoneal cavity were able to target the tumor site and reduce tumor size in mice. Lemon-derived vesicles enhanced the expression of apoptotic factors and reduced the concentration of pro-angiogenic factors such as VEGF-A, IL6, and IL8 in serum. These results suggested that the lemon EVs are able to reduce tumor growth in vivo, either by inducing TRAIL-mediated apoptosis, or by inhibiting the process of tumor angiogenesis [83]. Stanley and colleagues have recently provided confirmations of antitumor activities of Citrus-derived vesicles [74]. In particular, nano (NVs) and microvesicles (MVs) purified from orange, bitter orange, lemon, and grapefruit were demonstrated to specifically inhibit in vitro the proliferation of lung, skin, and breast cancer cells, with no substantial detrimental effects on the growth of non-cancer cells. Grapefruit-derived vesicles also suppressed G0/G1 and G2/M cell cycle transitions, promoted the upregulation of the cell cycle inhibitor p21, reduced the expression levels of various critical hallmarks of melanoma, such as those involved in tumor invasiveness and migration. These findings suggest that PDVs might be considered as co-adjuvants for the development of new therapeutic strategies to treat human malignancies.

### 5.6. Other Beneficial Effects of PDVs on Human Health

Exosome-like nanoparticles purified from nine vegetables or fruits (cilantro, aloe vera, grapefruit, garlic, turmeric, dandelion, lavender, cactus, and ginger) were selected to assess the inhibitory effects on activation of the NLRP3 inflammasome in primary macrophages [59]. Interestingly, among the PDVs tested, those obtained from ginger rhizomes strongly suppressed the pathways downstream of inflammasome activation such as caspase1 autocleavage, interleukin (IL)-1β and IL-18 secretion, and pyroptotic cell death.

Based on the above described promising results regarding the beneficial activities of nanovesicles purified from various edible plants, a few clinical trials started to verify the safety and establish the efficacy of PDVs. In particular, the properties of grape EVs and their ability to prevent oral mucositis as a side effect of chemotherapy treatment of head and neck cancers are being tested on humans (ClinicalTrials.gov Identifier: NCT01668849) and the capacity of EVs derived from aloe and ginger to reduce insulin resistance and chronic inflammation in patients who have been diagnosed with polycystic ovary syndrome (PCOS) (ClinicalTrials.gov Identifier: NCT03493984).

## 6. Bioengineering of PDVs to Boost Their Use in Nanomedicine

The ability to cross physiological barriers, often impermeable to other pharmaceutical vehicles, along with appealing pharmacokinetic and immunological properties make PDVs smart candidates to improve the delivery of therapeutic agents, with a primary interesting potential for the delivery of poorly soluble synthetic and natural biomolecules. While the biofunctionalization of artificial nanosized vesicular structures is well consolidated [84,85], the bioengineering of natural EV may represent a new challenge, but also an intriguing opportunity to develop personalized therapeutic carriers. In this section, we summarize the strategies thus far adopted to improve EV uptake in human cells, reporting the few examples developed for PDVs.

**Figure 5:**
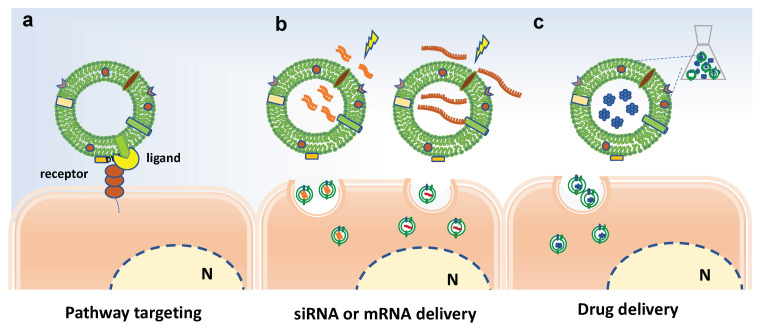
Bioengineering approaches to increase efficacy of plant-derived vesicles for therapeutic purposes. Schematic representation on current strategies and possible approaches for surface-functionalization and loading of extracellular vesicles. (**a**) Potential receptor binding ligands may target PDVs to specific cell population. (**b**) Electroporation to load miRNA and siRNA in PDVs. (**c**) Passive loading of bioactive molecules in PDVs. N indicates the nucleus.

Overall, the biofunctionalization of EVs aims at modifying their surface in order to improve the targeting properties of EVs or at introducing bioactive molecules or imaging tracers into EVs, as schematized in Figure 5. Basically, the surface modification can be achieved by genetic engineering or chemical modification [86]. For instance, chemical conjugation of small molecules to the surface of PDVs has be achieved through carbodiimide (EDC) crosslinker chemistry [87]. In particular, grapefruit-derived nanovesicles (GDNs) were EDC-conjugated with methotrexate (MTX), an immunosuppressant and anti-inflammatory agent, and successfully employed to treat acute colitis showing advantages, such as an increase in the therapeutic effects and a reduction of the side effects, in comparison to free MTX [82].

On the other hand, EV loading may occur though active or passive loading methods. The former involves temporary interruption of the membrane, through sonication cycles, freezing/thawing cycles or electroporation, followed by loading active compounds and restoring the integrity of the membrane. The latter involves the incubation of EVs with compounds able to pass through the membranes by passive diffusion, exploiting the concentration gradient.

The loading of nucleic acids in EVs often occurs by electroporation and subsequent incubation with EVs or alternatively by transfection-based approaches [88,89]. Recently, Zhuang and colleagues reported pursued a promising strategy to successfully load polyethylenimine (PEI)/miRNAs complexes in synthetic nanovesicles derived from grapefruits [90].

In addition to the use of plant EVs as a whole, alternative strategies have also proposed the use of lipids purified from EVs of edible plants to design new nanocarriers for the delivery of therapeutic agents. In particular, Wang and colleagues, in an original and pioneering work, demonstrated that nanocarriers derived from grapefruit EVs can deliver hydrophobic bioactive compounds (curcumin, folic acid, and Zymosan A), RNA, and proteins in a specific manner to different cell types, preserving the biological activity of the loaded molecules. The activity of these nanovectors has also been tested in vivo; in particular, they were loaded with the anti-Stat3 inhibitor JSI-12, an antineoplastic agent, and administered intranasally in mice with brain tumors. A more efficient reduction of the size of the lesions and an increase of survival of mice treated with functionalized nanocarriers compared to mice treated with free JSI-124 were observed, suggesting that the nanocarriers determine a better release of the drug in the tumor cells [91]. It is important to underline that in this study, the authors also proved that these nanovectors, when injected intravenously into pregnant mice, do not pass the placental barrier, validating the safety of this tools for drug delivery.

Finally, grapefruit-derived nanovectors were also shown to deliver therapeutic miR17 to brain. As mentioned above, polyethylenimine (PEI) and miRNAs were mixed, added to a lipid film extracted from grapefruit exosome-like nanoparticles, and then sonicated. These nanovectors were shown to inhibit the main brain tumor progress in mouse [90].

Similarly, nanocarriers made by lipids derived from ginger EVs were used as a carrier for the efficient delivery of the antineoplastic antibiotic doxorubicin (DOX) to colon cancer cells. Interestingly, ginger-derived nanovectors loaded with DOX were also surface-bioengineered by conjugation with folic acid in order to confer cell-specific targeting properties, since many tumor cells express a higher concentration of folate receptors. This work showed that DOX-loaded nanovectors have excellent biocompatibility and higher chemotherapeutic inhibition of tumor growth compared with free drug [80].

To increase the selectivity of drug release, grapefruit-derived nanocarriers, preliminary loaded with were therapeutic drugs such as curcumin or DOX, were coated with inflammatory-related receptor enriched membranes of activated leukocytes. Coated grapefruit-derived nanocarriers were able in vivo to specifically reach the site of inflammation and reduce symptoms of colitis in mice treated with DSS, as well as to significantly inhibit the growth of colon and breast tumor growth and extend the survival of tumor-bearing mice [92].

## 7. Conclusions

Pharmaceutical technology for several years has been directed to the realization of smart carriers for drug deliver. The review of the major current results and advances in this field, above reported and discussed, clearly indicate that plant-derived vesicles could be excellent carriers for the delivery of natural compounds and therapeutics. A series of intrinsic characteristics of PDVs are of instrumental importance to make them novel interesting tools for the delivery of natural bioactive compounds and drugs to specific cell targets, which are briefly summarized below:

- they are able to cross biological membranes and are biocompatible with mammalian cells;
- PDVs could solve most problems associated with the often reported toxicity or immunogenic and allergenic properties of synthetic nanomaterials, since it has been proved that most of them do not induce immune or inflammatory responses in normal host cells;
- they are stable and able to resist to the activity of digestive enzymes until they reach target cells, prospecting their use for oral administration;
- PDVs, presenting intrinsically bioactive natural compounds, could also deliver to tumor cells compounds with general detrimental effects, such as alkaloids and glycoalkaloids, cyanogenic glycosides, lectins, saponins, or other antinutritive factors;
- PDVs are unable to cross the placenta, and this last characteristic is very critical because it would allow the use of these vesicles to convey drugs, even potentially toxic ones, to pregnant women without repercussions on the fetus.

There are, however, major drawbacks and limitations in the pharmaceutical use of plant PDVs in nanomedicine due to the complexity of the extraction and the purification steps, and to the low rescue yields of purified EVs. Intensifying the basic research studies aimed at better understanding the biogenesis and the loading mechanisms of plant vesicles would surely help to overcome these problems. The identification of long-term storage strategies also appears to be critical to maintain the structural integrity and the bioactivity of PDVs once purified. Intensive integrated omics analyses are as well instrumental to pinpoint the distinct biomolecule profiles of the different classes of plant vesicles, thus allowing to design targeted therapy for different human diseases. In this direction, basic and clinical research on PDV bioactivities needs to be extended to a larger number of cell lines and mammalian models.

Finally, dissecting the biogenesis pathways and the genetic pattern controlling plant EV release may lay the foundation for designing genetic engineering approaches aiming at controlling and boosting EV production. Such innovative biotechnological strategies could lead to the production of plants able to secrete a greater amount of extracellular vesicles and/or to improve the compartmentalization of specific bioactive molecules in EVs. Another attractive goal is to engineer plants producing EVs expressing specific flags on their surface for precise cell targeting, thus promoting specific internalization and ameliorating their therapeutic activity.

## Figures and Tables

**Table 1:** Therapeutic effects of natural plant-derived vesicles purified from different fruits and vegetables. The table includes the plant species used as source of PDVs, the PDV size distribution, the biological activity observed, the experimental model employed to study the biological effects of PDVs together with the respective reference.

Species	Size	Biological Activity	Experimental Model	Reference
Grape	50–300 nm 500–1000 nm	oral mucositis protection of mice from dextran sulfate sodium (DSS)-induced colitis via induction of intestinal stem cells anti-inflammatory, anti-oxidative	colitis mouse model murine macrophage cell line (RAW 264.7) intestinal stem cells of Lgr5-EGFP-IRES-CreERT2 mice	*ClinicalTrials.gov Identifier: NCT01668849* [2,16]
Grapefruit	105.7–396.1 nm microvesicles (MVs 350–700) and nanovesicles (NVs 50–80 nm) 50–1000 nm	anti-inflammatory in vitro antineoplastic activity anti-inflammatory, anti-oxidative	C57BL/6 mouse model CD11b^+^F4/80^+^ lamina propria macrophages (LPMs) tumor cell lines (A375, A549, MCF3) murine macrophage cell line (RAW 264.7) intestinal stem cells of Lgr5-EGFP-IRES-CreERT2 mice	[2,74,82]
Ginger	102.3–998.3 nm 120–150 nm (rhizome) average size of ∼230 nm 90–1000 nm	liver protection inhibitory effects on activation of the NLRP3 inflammasome reduction of acute colitis, enhancement of intestinal repair, and prevention of chronic colitis and colitis-associated cancer (CAC) anti-inflammatory, anti-oxidative	mouse model alcohol-induced liver damage primary macrophages mouse colitis models murine macrophage cell line (RAW 264.7) intestinal stem cells of Lgr5-EGFP-IRES-CreERT2 mice	[2,59,61,79]
Carrot	85–110 nm 700–1500 nm	Anti-inflammatory, anti-oxidative	murine macrophage cell line (RAW 264.7) intestinal stem cells of Lgr5-EGFP-IRES-CreERT2 mice	[2]
Broccoli	18.3–118.2 nm	anti-inflammatory	BMDC-T cell co-cultures mouse colitis models	[45]
Limon	50–70 nm	in vitro and in vivo antineoplastic activity anti-oxidative	tumor cell lines (A549, SW480, LAMA84) NOD/SCID mice gastric cancer cell lines (AGS, BGC-823, SGC-7901) SGC-7901 tumor mouse model	[19,20,83]
Orange	50–150 nm	recovery of intestinal functions beneficial effects on metabolism and villi size	Caco-2 cells metabolic syndrome mouse model	[55]
Strawberry	30–191 nm	anti-oxidative	adipose-derived mesenchymal stem cells (ADMSCs)	[34]
Garlic	70–200 nm	anti-inflammatory	liver cells (HepG2 cell line)	[81]
Ginseng	Average size of ~344.8 nm	inhibition of melanoma growth	murine melanoma cell line (B16F10), breast cancer cell line (4T1) and human embryonic kidney cell line (HEK293T) melanoma mouse model (MyD88-, TLR4- and TLR2- deficient C57/BL6 mice)	[21]
Apple		Intestinal function	Caco-2 cells	[22]
Wheat	40–100 nm	Regenerative properties	primary human dermal fibroblasts (HDF; human endothelial vascular endothelial cells (HUVEC); human keratinocytes (HaCat)	[23]
Aloe and Ginger		reduce insulin resistance and chronic inflammation		*ClinicalTrials.gov Identifier: NCT03493984*

## Data Availability

No new data were created or analyzed in this study. Data sharing is not applicable to this article.
